# Building Safe Didactic Dialogues for Action Model: Mobilizing Community with Micronesian Islanders

**DOI:** 10.31372/20200501.1066

**Published:** 2020

**Authors:** Connie K. Y. Nguyen-Truong, Jacqueline Leung, Kapiolani Micky, Jennifer I. Nevers

**Affiliations:** 1 Dr. Nguyen-Truong and Dr. Leung are co-first authors.; a Washington State University College of Nursing in Vancouver, Washington, United States; b Micronesian Islander Community and Oregon State University, College of Public Health and Human Sciences in Global Health, United States; c Micronesian Islander Community; d Washington State University College of Nursing in Spokane, Washington, United States

**Keywords:** Micronesian Islanders, safe, didactic, dialogues, action, model, community-based, participatory, mobilizing community, educational innovation

## Abstract

*Background:* Despite mandates by the United States (U.S.) government to ensure the inclusion of women and minorities in federally funded research, communities of color continue to participate less frequently than non-Latinx Whites. There is limited research that examines maternal health outcomes and early childhood resources. Pacific Islanders (PI) have grown substantially in a county in the Pacific Northwest region of the U.S. (from 4,419 to 9,248, of which 52% are female). About 62.7% of PI women are not accessing prenatal care in the first trimester, and this is substantially higher than the national target of 22.1%. Researchers found that PI children are leaving school to take care of family obligations. The purpose of the educational innovative project, *Building Safe Didactic Dialogues for Action* model, was to respond to Micronesian Islanders (MI) parent leaders’ need to feel safe and to build a close kinship to encourage dialogue about difficult topics regarding access and utilization of early education systems and prenatal/perinatal health for community-driven model for action planning and solutions. *Approach:* Popular education tenets were used in the project to be culturally sensitive to the human experience. The MI community health worker outreached to MI parent leaders in an urban area in the Pacific Northwest region of the U.S. Eight partners participated in this project: parent leaders from the MI community-at-large, community partners from the MI Community organization, and academic nurse researchers. Didactic dialogues lasted two hours per session for four. Topics included: collaborative agreements, MI parent leaders’ identified needs and existing resources regarding preschool and immunization, parent–child relationship (stress and trauma, adverse childhood experiences), and MI experience regarding prenatal care access and postpartum depression. Group discussion on reflection was used to evaluate utility. *Outcomes:* *Building Safe Didactic Dialogues for Action* model was foundational via mobilizing community with MI, trust and rapport building, and engaging in a safe and courageous space for dialogues for action planning and solutions as community and academic partners as stakeholders. *Conclusion:* Many previously unspoken issues such as abuse, language, and cultural beliefs including barriers were openly shared among all partners. Dynamic thoughts towards identifying needs for change and then planning steps toward creating positive change created an atmosphere of empowerment for change.

There are historical and rising trends with culturally insensitive research methodologies, interactions, and implementations of projects in several diverse communities (Bozzo, Bali, Evaniew, & Ghert, 2017; Centers for Disease Control and Prevention, 2015; Li et al., 2018; Pobutsky, Buenconsejo-Lum, Chow, Palafox, & Maskarinec, 2005; Skloot & Turpin, 2010). Trust, rapport, and safety in dialogues are important for sustainable relationships with communities of color who have suffered historical trauma (Nguyen-Truong, Closner, & Fritz, 2019; Nguyen-Truong et al., 2018). To understand community perspective from a culturally sensitive lens and to build dialogue for community-driven action planning and solutions, academic partners must build rapport and trust within the community.

Micronesian Islanders (MI), a subgroup within the Pacific Islander group, have an immense amount of health disparities (Marshall, 2006; Pobutsky et al., 2005); however, research tends to focus on health conditions such as chronic diseases including diabetes, obesity, and cancer. There is a limited amount of research that examines maternal health outcomes and early childhood resources, and findings point to critical needs in the Pacific Islander group. In a county report where Pacific Islanders have grown substantially in the Pacific Northwest region of the United States (U.S.; grew from 4,419 to 9,248, of which 52% are female), 62.7% of Pacific Islander women are not accessing prenatal care in the first trimester, and this is substantially higher than the national Healthy People 2020 target of 22.1% (Multnomah County Health Department, 2015). In addition to Pacific Islander women being less likely to access prenatal care during their first trimester than non-Latinx White women (62.7% versus 24.3%, respectively), they are also more likely to have low birthweight babies (less than 2,500 grams) than non-Latinx White women (9.3% versus 5.9% respectively; Multnomah County Health Department, 2015). In regards to childhood, researchers found that although Pacific Islander children want to learn and want to be successful, but they are connected to their Pacific Island culture where if there is a family obligation, then children will leave school and have high absentees or drop out to take care of family such as caring for an ill family member (Ratliffe, n.d.). About 58.8% of Pacific Islanders who are 25 years or older have no more than a high school education versus 27% of non-Latinx Whites (Multnomah County Health Department, 2015).

MI may be reluctant and wary to participate in research because of unethical research conducted in the Pacific (George, Duran, & Norris, 2014; Scharff et al., 2010). One of the large harmful impacts was when MI experienced diaspora, being forced to leave the homeland. The U.S. government tested nuclear bombs in a region of Micronesia that destroyed the ecological systems on the island and surrounding territories, and there was forced permanent relocation of communities that the bombs were dropped on (Pobutsky et al., 2005). As a result, many MI have trust issues with the U.S. government.

MI consist of multiple countries and islands. For purposes of our project, we focused on MI from the Federated States of Micronesia, the Republic of Marshall Islands, and the Republic of Palau. MI from these countries are Compact of Free Association (COFA) citizens. The Compact of Free Association is an international agreement that established and governed the relationship between the U.S. government and the three Pacific Island nations in 1986. Under the compact, the U.S. promised to provide financial assistance over a 15-year period in exchange for full international defense authority and responsibilities including the building of U.S. military bases on their islands. Further, COFA citizens were promised medical care and other benefits, and were permitted to travel freely to the U.S. states and territories without a visa (only needing a valid passport and I94), while being legally allowed to live, work, and attend school (McElfish, Hallgren, & Yamada 2015). However, in 1996, the signing of the Personal Responsibility and Work Opportunity Reconciliation Act (PRWORA) of 1996 resulted in COFA citizens no longer being qualified for these prior promised benefits (McElfish et al., 2015). Engagement with community and academic partners as stakeholders that build safe didactic dialogues for community mobilization in response to community identified needs while building trust enables honest conversations on community-driven action planning for solutions.

*Building Safe Didactic Dialogues for Action* model (hereafter referred to as *Building Safe DDA*) is an educational innovation project. The purpose was to design, implement, and evaluate the project to respond to MI parent leaders’ need to feel safe and encourage dialogue through trust building activities centered on difficult topics including early education systems and prenatal/perinatal health care within the MI community-at-large for community-driven model action planning with the Micronesian Islander Community, a culturally diverse community-based organization, and nurse researchers at Washington State University College of Nursing, a large public university in the Pacific Northwest in the U.S. The MI parent leaders in our project are trusted community members and identified as being mothers in the MI community-at-large and expressed that MI is a *shy and humble culture* not accustomed to speaking about community issues such as access to early education systems and prenatal/perinatal health care, while community organizing towards leading a community-driven solution. *Building Safe DDA* is a model and was a foundation mobilizing effort to build community with MI, trust and rapport building, and engagement in a safe and courageous space for dialogues for action planning as community and academic partners as stakeholders. In this article, we described the design, implementation, and evaluation of *Building Safe DDA* as an innovative educational model method.

## Approach

### Design

#### Pedagogical approach

We used Popular Education tenets (i.e., empowerment) to be culturally sensitive to the human experience (Wiggins, 2012). Tenets included the following: learning with our heads (i.e., cognitive), hearts (i.e., affective), and bodies (i.e., psychomotor); creating an open and trusting environment for learning where community and academic partners can share their perspectives and experiences; promoting equality between partners as stakeholders; validating knowledge acquired through life experience is equally important as knowledge acquired through formal education; actively participating in learning; and using artistic teaching as important tools for mobilizing community (Nguyen-Truong et al., 2018; Wiggins, 2012). Group reflection of the MI parent leaders, community partners from the Micronesian Islander Community (MIC) organization, and academic nurse researchers (collectively referred to as partners) occurred throughout *Building Safe DDA* model and was used for evaluation of the group interaction and responses regarding the utility of this educational method.

Furthermore, partners engaged in ongoing self-reflection and debriefed about safety within the context of culture, ensuring awareness of the importance of being culturally sensitive to the historical trauma within the MI community. We considered several important cultural safety tenets. This includes *knowing* one’s self; how one’s history/background contributes to values, bias, and attitudes toward others; and walking alongside one another (Nguyen-Truong et al., 2019; Nursing Council of New Zealand, 2011). Community and academic partners worked together to build a safe and welcoming environment while developing self-reflection activities that encouraged dialogue while building rapport with one another.

### Implementation

#### Setting and Community and Academic Partners as Stakeholders

The Washington State University Office of Research Assurances has determined that the project (IRB # 17203-001) satisfies the criteria for Exempt Research at 45 CFR 46.101(b)(2) and found that the project is exempt from the need for Institutional Review Board review. *Building Safe DDA* model was carried out with community and academic partners at a community organization conference room for two hours, one session per month for four months in an urban area of the Pacific Northwest region of the U.S. The meeting format was created based on the request to meet the MI parent leaders’ availability.

#### Underpinnings of the Process in Building Safe DDA

**Figure 1.**
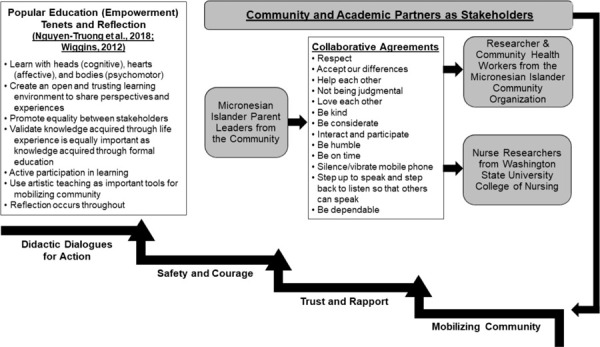
Overview of building safe didactic dialogues for action model.

See Figure 1 for an overview of *Building Safe DDA* model that consisted of four key pillars: mobilizing community, trust and rapport, safety and courage, and didactic dialogues for action planning.

##### Mobilizing community

MI parent leaders from the MI community-at-large, community partners from the MIC organization, and academic nurse researchers partnered as stakeholders on the shared value of collective empowerment. This is a foundational step. The MIC organization focuses on social justice, the preservation of MI culture, and provides opportunities for leadership through unifying diverse communities within the MI community. Due to MI parent leaders’ distrust of the government as described earlier, a MI community health worker (CHW) staff who is bilingual and bicultural and identifies as a parent of young and adolescent children outreached to the MI parent leaders regarding this project. The MI parent leaders were receptive to engage with the MI CHW as the staff member was someone they knew and trusted from the community-at-large.

According to the American Public Health Association (2019), “A community health worker is a frontline public health worker who is a trusted member of and/or has an unusually close understanding of the community served and serves as a link between health/social services and the community to facilitate access…” First the MI CHW introduced one’s self as Chuukese and talked briefly about one’s family as to connect with MI parent leaders for a comfortable conversation. The CHW described their role as a CHW, including conducting home visits with families while providing support in identifying and obtaining access to needed resources for families with children 8 years old and younger. Further, the CHW welcomed contact by telephone or email and provided a business card with their contact information. These activities lead to building trust when MI parent leaders called the CHW for appointments for assistance to resources. At the appointment, the CHW talked about the opportunity to participate in the project as a partner.

The Executive Director/a primary researcher/CHW from the MIC organization and academic nurse researchers have a standing relationship through their prior research collaboration with another population on infusion of the empowered voice in design, implementation, and analysis (Asian immigrants). Thus, a total of eight partners participated, of which four were parent leaders from the MI community-at-large (from the Federated States of Micronesia and Republic of Marshall Islands), the Executive Director/a primary researcher/CHW and one trained MI certified CHW (from the Federated States of Micronesia) were from the Micronesian Islander Community, and two academic nurse researchers. We provided a meal and quality childcare for partners to address identified barriers to participation in this project. MI parent leaders each received a $25 stipend per session as renumeration.

##### Trust and rapport

The first session was foundational to trust and rapport building for a comfortable interactive environment. Each partner introduced self, their role, and why they were there. The MIC organization staff and an academic primary nurse researcher reviewed the *Building Safe DDA* model as a part of the Health and Education program. Partners from the MIC organization and the academic nurse researcher asked MI parent leaders to develop collaborative and collective agreements on how they envisioned the group to interact within the sessions as the next step. This promotes discussion and transparency of expectations among partners including values for building rapport and trust. MI parent leaders presented their proposed list of collaborative agreements that was mutually agreed upon by and adopted by all partners (see Figure 1). We visited these agreements at the start of each session to uphold for ongoing trust and rapport building and would add to it. Following the first session, partners added “*step up to speak*” and “*to step back to listen so that other can speak,*” and “*being dependable*” to encourage partners to engage in dialogues that was sensitive to MI parent leaders, taking into account that the parent leaders identified as being a shy and humble community.

##### Safety and courage

Community partners’ role from the MIC organization and academic nurse researchers’ role included being trusted partners and as facilitators, though not as teachers to MI parent leaders as parents were viewed as community experts. This distinction is important because community partners from the MIC organization and academic nurse researchers co-created a safe and courageous space for MI parent leaders to develop their voice and engage as a collective versus passive observers.

MI parent leaders reported community issues of importance that they would like to work as partners with for action planning. MI parent leaders missed their island homes and struggled in maintaining MI cultural identity, while adjusting to western life in the U.S. Specifically, MI parent leaders struggled with learning and understanding English language and culture. The partners discussed and agreed upon community issues including the importance of childrearing and child development struggles with their children born on the islands and in the U.S. Thereby, the three main areas of concern within child development included delaying prenatal/perinatal care, access to quality and affordable preschool, ensuring proper immunizations, and the dynamic of the parent–child relationship, especially as their children become accustomed to U.S. culture and values.

The *Building Safe DDA* model was conducted in English based on preference of the MI parent leaders. The trained MI CHW conducted visits with MI parent leaders outside of the monthly sessions to support safe DDA. The MI CHW worked with MI parent leaders on their presentations and prepared the group for facilitation to encourage confidence in public speaking. The MI parent leaders were separated into two diverse groups. One group expressed interest in exploring the early learning systems such as preschool and early head start, given their children are in preschool and their personal experiences in dealing with issues related to the early learning system. The second group decided to focus on the delay in prenatal care. The personalized visits encouraged MI parent leaders to work closer together to speak on issues that were important to their communities and to develop ideas/programs that would support reaching their goals. During these visits, MI parent leaders documented specific goals, initially identified existing resources, and an overview on an implementation plan, onto white flip chart papers. The documents were later displayed on the wall at the sessions.

Academic nurse researchers prepared for facilitation of the parent–child relationship session that focused on stress and trauma. MI community partners from the MIC organization reviewed the content on neurobiology of stress and trauma and adverse childhood experiences (ACEs), linked to health diseases as adults for cultural and language appropriateness and responsiveness to the needs of the MI community. The academic nurse researchers incorporated the MI community partners’ recommendations from their review including the reduction of clinical terminology, the addition of historical and recent MI issues including the history of nuclear bomb testing in the Micronesian coastal lands and Typhoon Wutip. Through small and large group reflection exercises, the MI parents shared personal, family, and MI community experiences and their perceptions and considerations regarding the parent–child relationship.

##### Didactic dialogues for action planning

Didactic dialogues focused on MI parent leaders’ identified community needs and existing resources to plan for implementing change regarding preschool attendance and immunization, strengthening the parent–child relationship (the impact of stress and trauma including ACEs), and preventing the delay of prenatal care. During the sessions, some MI parent leaders on occasion presented with long pauses or silence having to process their thoughts, or in some cases, expressed shyness including being women and speaking openly as it was not common to do so within the MI culture. The community and academic partners encouraged MI parent leaders to feel comfortable in their presentation and provided support in speaking their MI language. Supportive statements included “Would it help if you said it in your language?”, “Is there an equivalent word or phrase in your language?”, and “How would you say it in your language?”. Some parent leaders elected to present in their own language, with another parent leader interpreting in English. The trained MI CHW is a qualified interpreter and verified the interpretation.

### Educational Project Evaluation

The Executive Director/a primary researcher/CHW, the MI CHW, and an academic primary nurse researcher co-facilitated one session. MI parent leaders co-facilitated two sessions, and two academic nurse researchers co-facilitated one session. The trained MI CHW and two academic nurse researchers co-facilitated the group discussion and documented field note based data including group interaction and evaluation on responses regarding utility and verified content and interpretation with all partners to ensure trustworthiness (Lincoln & Guba, 1985). Community and academic partners provided their perceptions and experiences on the utility of the *Building Safe DDA* model. The Executive Director/a primary researcher/CHW, the MI CHW, and the academic nurse researchers met afterwards and debriefed on the field note based data.

## Discussion on Outcomes

### “Struggle is Real for MI Children”

#### A Need to Visualize the Importance of Early Learning

The MI parent leaders worked with the MI CHW and discussed Head Start as an early learning system (specifically preschool) of focus. According to the U. S. Department of Health & Human Services (n.d.), Head Start is designed to help stop the cycle of poverty that provides children of low-income families with a comprehensive educational and social program to meet their emotional, social health, nutritional, and psychological needs. Pacific Islander children are more than twice as likely to experience poverty as non-Latinx White children (Multnomah County Health Department, 2015). Micronesian Islanders as a subgroup within the Pacific Islander group should be recognized among the uninsured, lowest education, English as a second language, and foreign-born (Kataoka-Yahiro, 2017). The initial conversation included immunization requirements for children (or documentation of exemption) to be able to attend preschool.

Through discussions as partners, there is a critical need to outreach to parents and families in the MI community-at-large on the importance of preschool to children, to increase enrollment of MI children in early learning programs, and in doing so, promoting the immunization rate for MI children. MI parent leaders identified the following goals for families and children regarding the intersection of early learning and health. Goals for MI families included addressing family well-being, encouraging the parent–child relationship, including the family as learners, and supporting family as advocates and leaders. Goals for children included ensuring readiness for school (e.g., kindergarten), safety and health, and ensuring properly aged child development and learning occurring.

The *Building Safe DDA* model helped all partners delve deeper into MI community issues related to these goals. MI parent leaders expressed concern that most MI families do not consider preschool an important academic milestone and would often wait until a child is 5 or 6 years old before enrolling them into kindergarten. For example, a MI parent leader described, “*The struggle is real for MI children*” regarding “*falling behind the other children in the general population such as not knowing the alphabet, not understanding what other children or other adults are talking about, being left out and feeling alone, and [feeling] like a stranger in the classroom.*” This adds to the literature. According to the AARP (2014), (42)% of Asian Americans and Pacific Islanders are providing care for older adults versus 22% of the general population. The following sheds light. Although 30% of Asian Americans and Pacific Islanders reported that they had adult children helped them in taking care of older family members, but there was not specific mention about younger children helping to take care as well (AARP Executive Summary Report, 2014). Prior researchers found that Pacific Islander children would leave school to take care of family obligations such as caring for a family member who was ill (Ratliffe, n.d.). AARP (2014) underscored that younger family members may tend to older adults with severe illness to make older adults comfortable for the rest of their lives rather than help older adults to return to full functioning and independence.

MI parent leaders talked about wanting to use testimonies from MI families who have or have had children in preschool to help other MI families visualize the importance. They also discussed about specific need for an Islander focused component in preschool to enhance connections between MI families and their children’s teachers.

### Defining and Describing: When Stress and Trauma are Not Words

Academic nurse researchers led a dialogue on stress and trauma to be culturally responsive to MI community partners’ (from the MIC organization and MI parent leaders) need regarding considerations in the parent–child relationship. This bridged a critical conversation between academic and community partners as there were no equivalent word for stress and trauma in MI languages. All partners were encouraged by academic partners to engage with the shared information through co-constructing the dialogue. This was an authentic collaborative live process designed to welcome participation instead of passive learning, while ensuring partners are comfortable during the sharing process.

An academic nurse researcher shared information that included images that progressed from the general underpinnings on neurobiology of stress and trauma to specific issues relevant to the MI community. Community partners openly shared their thoughts and experiences in regard to these issues. Community partners described a strong familiarity with images of the U.S. nuclear bomb testing after World War II and the impact on the environment and life in Micronesia (images from Bettman Getty, n.d.; Scott, 2017). Community partners mentioned that some islands are more crowded with residents due to the inability to live in areas of high radioactivity. The nuclear bomb testing served as an example of historical trauma due to human made events, and the 2019 Typhon Wutip (Cappuci, 2019) served as an example of current trauma created by nature. A community partner from the MIC organization stated her husband, “…*the Chief of the Chuuk Island of Micronesia, was tearful and very worried about the impact on his community in Micronesia and that he failed his people.*” This partner further described feeling very concerned about her husband and the community members of the island as “*many people are still homeless and are living in huts* [a house made of coconut leaves] *until their homes are rebuilt* [in the island].” The message all partners took away is that external traumatic events (whether caused by humankind or by nature) will have a major impact to the community and individuals and the impact may be lifelong.

All partners also discussed considerations regarding sensory triggers of trauma. Most partners described experiences with reliving trauma as triggered by sensory input. A community partner from the MIC organization described triggers to connect to this notion. This partner shared an olfactory sensory trigger of the scent of vanilla and auditory trigger of hip-hop music that developed during a stressful time during her pregnancy. Although her pregnancy occurred several years ago, her biological response to current encounters with the scent and genre of music still evoke a powerful emotional response such as being upset and having a physical response (nausea), respectively.

MI parent leaders described that during pregnancy, they knew of MI women who developed an intense aversion to the scent and the appearance of the father. This led women to avoid further contact with the father during their pregnancy. A community partner from the MIC organization described an experience of being triggered by auditory cues to specific words during a past traumatic event. During the triggered experience, this partner did not understand why she cried because the auditory cues did not consciously stir-up memories of the past trauma. All partners expressed similar experiences of auditory and olfactory triggers. All community partners expressed that knowledge and comfortable dialogue of triggers, signs, and symptoms of stress/trauma was empowering, while validating lived experiences previously not acknowledged within the scope of MI languages and cultures.

### Speaking the Unspoken: Silence of Love and Need to be Heard

An academic primary nurse researcher partner discussed a video with all partners about the long-term impact of ACEs and how ACEs are based on the numbers of ACEs (Burke Harris, 2014) and encouraged all partners to have in-depth reflection about what ACEs mean to the group and in the MI community. ACE is a term used “to describe all types of abuse, neglect, and other potentially traumatic experiences that occur to people under the age of 18,” and ACEs are linked to risky health behaviors, chronic health conditions, low life potential, and early death (Centers for Disease Control and Prevention, 2019, para. 1). A community partner from the MIC organization shared that there is no equivalent word for ACEs in MI languages. All community partners were concerned about the minimal degree of physical and verbal affection between parents and their children in the MI culture. An additional concern related to language was not having the equivalent words to verbally express “*I love you*” to their children. A community partner from the MIC organization shared that parents typically show their love for their children by feeding and taking care of them, and not through the use of words. All partners asserted that children need to hear their parents tell them they are loved.

Some MI parent leaders and community partners from the MIC organization shared their experience in witnessing or personally experiencing childhood abuse as a child in Micronesia, including physical and sexual abuse. There was a pervasive sense of helplessness and fear of talking about what was happening around them as children. Pacific Islanders who were survivors of child sexual abuse reported concerns for the family and self-blame as the most common reasons for delay and not disclosing about the abuse (Xiao & Smith-Prince, 2015). Prior researchers examined the associations between parenting including parent–child relationship satisfaction regarding love and various subsequent children psychosocial, mental, behavioral, and physical health and well-being outcomes (Chen, Haines, & Charlton, 2019). Chen et al. (2019) found that greater relationship satisfaction was associated with greater emotional well-being, lower risk of mental illness, eating disorders, overweight or obesity, and marijuana use. In other studies, Pacific Islander parents reported feeling pressure to love their children through physical discipline. The failure to instruct and if necessary, physically discipline their children is viewed as a failure to love their children and hitting is considered an act and a duty to show love (Fairbairn-Dunlop, 2001). This is an important consideration regarding Pacific Islander communities where adults hide expressions of love to prevent their children from becoming spoiled, while in return, children learn to respect and obey their parents in return (Gerber, 1985). The discussion with MI parent leaders and community partners from the MIC organization led to an emphasis on a need to broadly reach MI members in the community to improve the connection in parent–child relationships and to encourage the expression of love, while encouraging safe dialogue between parents and children.

### Cultural Beliefs and Practice: Prenatal Care, Delivery, Postpartum Care

MI parent leaders and a community partner from the MIC organization expressed concern about MI mothers including adults and adolescents and the delay in prenatal care. As stated by a MI parent leader, “*Don’t talk about prenatal. Don’t know until you experience it* [referring to later in the pregnancy].” MI parent leaders shared that community members do not see the importance of prenatal care. As described by another MI parent leader, “*Do we have to go? Don’t know about ultrasound and needing to know the position of the baby. Back in the island, they massage* [referring to midwife who are women who have had experience with birth support and is usually an elder who was trained by experience]. *The midwife knows and will reposition….*” A community partner from the MIC organization described the fear of approaching the topic about sex with her parents when she was young. “*Mom don’t talk to us about sex. I was afraid. If ask, then afraid they said why you want to know, to do something bad?*” MI parent leaders described how different the care is for MI mothers in the island compared to the U.S. as “*Everyone* [referring to MI]* wants to use island technique and not U.S. technique*” and it is “*laid back in the island.*” For example, the only form of support in the island is from families and a midwife, whereas, there are additional resources in the U.S. such as the free clinic or medical offices for regular check-ups.

MI parent leaders shared that medications are limited on the island and involves shipping from elsewhere to the island. As such, there is heavy reliance on cultural rules and home remedies such as observing the island spirit, chanting to help with bearing a baby, and local medicines found naturally on the islands. For example, MI parent leaders described how pregnant women cannot go out when its dark or they will get possessed by these spirits that will result in being unable to bear a baby: [shared by a Chuukese parent] “*During pregnancy, the women are not supposed to walk outside when it is dark and raining.*” There is a belief that women who walk outside when it is dark and raining will be harmed by spirits and will have a difficult delivery. This community partner from the MIC organization also described signs that “*the spirits have already harm them,*” usually by physical changes to their body. The partner described it as “*a change in skin color to very dark*” or “*black on their body*” or parts of their body. The mom or auntie of the pregnant woman will give her local medicine such as an oil to put on her body or to eat or drink the local medicine in order for the pregnant women to have a relaxing and easy labor.

Although there are cultural beliefs and traditional practices that MI uses as early care and intended to be protective, but there appears to be a reliance on physical signs. This can mean delay in seeking and receiving prenatal care in a healthcare system and underscores the importance of this culturally sensitive need in reaching to MI early in the pregnancy. According to the National Council for Asian Pacific Islander Physicians (2018), although a healthcare provider does not need to conduct a comprehensive health needs assessment of social and behavioral determinants at the first visit or all in one visit, but a healthcare provider should set aside time to discuss more sensitive needs with clients. Traditional practices tend to lean toward avoiding healthcare providers; therefore, Pacific Islanders tend to visit a healthcare provider less frequently and not take medicines prescribed for the long term (AARP Executive Summary Report, 2014). Prior researchers found that MI mothers experienced several structural and socio-cultural barriers that constrain prenatal care. Structural barriers included access to health insurance, transportation, and language barriers, and socio-cultural barriers included not understanding the importance of seeking early and having consistent prenatal care and how to navigate the healthcare system (Ayers et al., 2018).

There is a belief that during delivery means between death or life and carries risk. Thus, family members and the community through social networks come and sit around during labor and delivery to give support to the pregnant women. MI parent leaders and a community partner from the MIC organization described labor and delivery in the U.S. and in the island. In the U.S., a majority of the family members who are close to the pregnant women will come and sit with the women or will wait in the lobby to give support during the labor time. However, only women are allowed to come close to the pregnant women during delivery. Males are expected to sit at a distance or outside of the room. In the island, families will wait in the lobby to give support but are not allowed to go in with the pregnant women during labor and delivery because of the small rooms.

MI parent leaders and a community partner from the MIC organization delved into depth about cultural beliefs passed from generation to generation regarding spirits for postpartum care for the Chuukese and Marshallese. There seems to be similarities and differences among these subgroups that adds to the literature. For the Marshallese, there is a cultural belief that when a woman gives birth, they “*have to take the placenta and kill it.*” This involves taking the placenta, pounding on it or tearing it apart until they feel the placenta is dead, then bury the placenta in their backyard. In doing so, the placenta will not come back to harm them. However, not engaging in this cultural practice could result in the mother and child being harmed by the spirits of the placenta. After giving birth, the women are expected to bathe in coconut oil, hot water, and place leaves in their vagina as a local medicine to facilitate healing and gaining strength. For the Chuukese, after the delivery, the family must take the placenta and bury it where they call home or in a land that belongs to the family in order for the women to have an easy pregnancy and labor if they become pregnant again. The women also bathe in coconut oil and turmeric, and put leaves wrap in cus before being placed in the vagina to facilitate healing and strength. During the healing time of the mother and baby, the father or husband cannot come close and must sleep in another house until the baby turns 1 year old. Although MI parent leaders and a community partner from the MIC organization expressed respecting and honoring the cultural beliefs and practices, they discussed that men need to become more involved with the pregnancy and postpartum healing process of the mother and infant. Prior research findings in New Zealand point to the importance of parenting including the role in developing healthy families and children. In particular researchers found that Pacific Islander fathers felt their role is not valued by society, yet they are raising the citizens of the future (Families Commission, 2007). Pacific Islander fathers reported high levels of involvement with their children and increased father involvement was significantly associated with a lower risk of child behavior problems (Families Commission, 2007). However, Pacific Islander fathers with less affinity with their traditional Pacific Island culture exhibited lower levels of father involvement compared to fathers with strong affinity with their Pacific Islander culture (Families Commission, 2007). This helps to shed light on the need to examine how Pacific Islander fathers may feel as appreciated as a parent to their children.

## Limitations

A limitation is that *Building Safe DDA* focused on a limited number of MI community members. Cultural expectations in safe creation of didactic dialogues for action planning may be different in other groups. This educational innovation project was carried out in an urban area in the Pacific Northwest of the U.S.

## Recommendations

Important considerations include community identified needs may be different in other racial-ethnic groups and for communities in other urban and rural geographic locations. Other important considerations include whether years residing in the U.S. impact action planning, whether the MI is a COFA citizen, U.S. Citizen (as in Commonwealth of the Mariana Islands and Guam), and the gender of the MI participants.

## Conclusion

The *Building Safe DDA* model provided a cultural lens into the lived experiences of MI parent leaders and community partners from the MIC organization. Through collaborative efforts between the MIC organization and the academic nurse researchers, we were able to partner with MI parent leaders, resulting in positive growth and development within the community. The work resulted in building trust and collaboration between MI parent leaders, the MIC organization, and academic nurse researchers. The community and academic partners spoke of the value of stress reduction for parents and making cultural changes to decrease potential childhood stress. The group dynamic encouraged safety to discuss sensitive issues. Many previously unspoken issues such as abuse, language, and cultural beliefs including barriers were openly shared among all partners. Dynamic thoughts toward identifying needs for change and then planning steps toward creating positive change created an atmosphere of empowerment for change. MI parent leaders have developed positive, uplifting, and valuable leadership and community organizing skills to begin the next stage of the project—becoming informed, engaged, and valued community leaders with community organizing and public speaking skills.

## Acknowledgments

The authors are appreciative of the anonymous peer reviewers for assistance.

## Declaration of Conflicting Interests

The authors declared no potential conflicts of interest with respect to the research, authorship, and/or publication of this article.

## Funding

Dr. Jacqueline Leung, JD, MS, CHW, Dr. Connie Kim Yen Nguyen-Truong, PhD, RN (Alumnus PCCN), and Kapiolani Micky, BA, CHW, received the Health and Education Fund’s Impact Partnership #18-02376 that supported the project: Northwest Health Foundation, Care Oregon, Kaiser Permanente Northwest, Meyer Memorial Trust, and Oregon Community Foundation.
